# Role of the *EGF *+61A>G polymorphism in melanoma pathogenesis: an experience on a large series of Italian cases and controls

**DOI:** 10.1186/1471-5945-9-7

**Published:** 2009-07-22

**Authors:** Milena Casula, Mauro Alaibac, Maria A Pizzichetta, Riccardo Bono, Paolo A Ascierto, Ignazio Stanganelli, Sergio Canzanella, Grazia Palomba, Edoardo Zattra, The Italian Melanoma Intergroup (IMI), Giuseppe Palmieri

**Affiliations:** 1 Unit of Cancer Genetics, Institute of Biomolecular Chemistry, Consiglio Nazionale delle Ricerche, Sassari, Italy; 2 Department of Dermatology, University of Padua, Padua, Italy; 3 Division of Medical Oncology, Centro di Riferimento Oncologico, Aviano, Italy; 4 Department of Dermatology, Istituto Dermopatico dell'Immacolata, Roma, Italy; 5 Unit of Medical Oncology and Innovative Therapy, National Cancer Institute Fondazione Pascale, Napoli, Italy; 6 Dermoscopy Unit, Tumor Institute Romagna, Meldola, Forli, Italy; 7 Unit of Skin Cancer Prevention, Association House Hospital Onlus, Napoli; Italy

## Abstract

**Background:**

A single nucleotide polymorphism (61A>G) in the epidermal growth factor (*EGF*) gene has been implicated in both melanoma pathogenesis and increased melanoma risk. To further evaluate this association, we conducted a case-control study in a clinic-based Italian population.

**Methods:**

Individuals with less than 10 (N = 127) or more than 100 (N = 128) benign nevi, and patients with cutaneous melanoma (N = 418) were investigated for the *EGF *+61A>G polymorphism, using an automated sequencing approach.

**Results:**

Overall, no difference in *EGF *genotype frequencies was observed among subjects with different number of nevi as well as when non-melanoma healthy controls were compared with the melanoma patients. However, a heterogeneous distribution of the frequencies of the G/G genotype was detected among cases and controls originating from North Italy (21.1 and 18.3%, respectively) vs. those from South Italy (12.6 and 17.1%, respectively).

**Conclusion:**

Our findings further suggest that *EGF *+61A>G polymorphism may have a limited impact on predisposition and/or pathogenesis of melanoma and its prevalence may vary in different populations.

## Background

The epidermal growth factor (*EGF*) gene, which is a member of the EGF superfamily, has been demonstrated to activate cell proliferation and stimulate mitogenesis in epidermal tissue, enhancing tumour growth [1].

In a previous case-control study conducted in the United Kingdom, the 61A>G transversion (rs4444903) in the *EGF *gene has been correlated with an increased risk of melanoma *in vivo *[2]. In particular, the prevalence of the G/G genotype was significantly higher in melanoma patients than in healthy individuals as controls (both originating from northern European countries); the G allele was present in nearly 66% of patients with malignant melanoma (odds ratio 4.9 [95% CI 2.3–10.2]; p < 0.0001) [2]. Overall, the A and G alleles have been reported in 56–63% and 37–44%, respectively, of all the *EGF *alleles in the European Caucasian populations [2-8]. Among melanoma patients, a significant association with the Breslow thickness was also inferred (odds ratio 3.7 [95% CI 1.0–13.2]; p = 0.045) [2].

From the biological point of view, it has been hypothesized that presence of the G allele at position 61 of the *EGF *gene leads to increased *in vitro *expression of the EGF protein (suggesting that such a sequence variant may act as a functional polymorphism) [2]. Since the original report, majority of studies conducted in different Caucasian populations have not confirmed such an association between the *EGF *+61A>G polymorphism and the predisposition to melanoma or nevi development [3-6]. Moreover, conflicting findings have been also reported about the correlation of such a polymorphism with Breslow thickness and survival prediction [4-7].

Through a case:control study carried out within the entire Italian population, we here investigated the role of the *EGF *+61A>G polymorphism in nevi formation and melanoma pathogenesis, in order to further assess whether the G allele-dependent EGF production might be important in the development of such a disease.

## Methods

About 1,500 individuals underwent a whole-body skin examination using the epiluminescence microscopy. Melanocytic nevi were found in the range between 1–9 and 10–100 in about 23% and 68% of the participants, respectively; about 9% of them had more than 100 benign melanocytic nevi. One hundred and twenty-seven consecutively-collected individuals with low number of benign melanocytic nevi (<10), 128 subjects with high number of benign melanocytic nevi (>100), and 418 patients with histologically-proven diagnosis of cutaneous melanoma were included into the study. To avoid any bias, melanoma patients were consecutively collected from January 2003 to December 2005; they were included regardless of age at diagnosis, family history status, and disease features. Histological classification (including Breslow thickness and Clark level of invasion) was confirmed by medical records, review of pathologic material, and/or pathology reports.

Patients were from different Italian regions; non-melanoma healthy controls aged 21–60 years (either those with high or those with low nevi numbers) were chosen as representative of the individuals living in the same geographical areas and were comparable for sex, age and general phenotype (hair and eye color, tanning type) to patients with melanoma. After individuals were informed about aims and limits of the study, blood samples were obtained with their written consent. The study was reviewed and approved by the ethical review boards of both the Azienda Sanitaria Locale of Sassari-Olbia and the University of Sassari.

Genomic DNA from all cases and controls was isolated from peripheral blood, using standard methods, and then screened for the A>G polymorphism at position 61 of the 5' untranslated region of *EGF *gene, using an automated direct sequencing approach. Primers for polymerase chain reaction (PCR) assays were as previously described [2].

Odds ratios of carrying the *EGF *+61A>G polymorphism were estimated by the logistic regression model and reported with 95% confidence interval (95% CI). Analyses were performed with the statistical package SPSS/7.5 for Windows.

## Results and discussion

**Figure 1:**
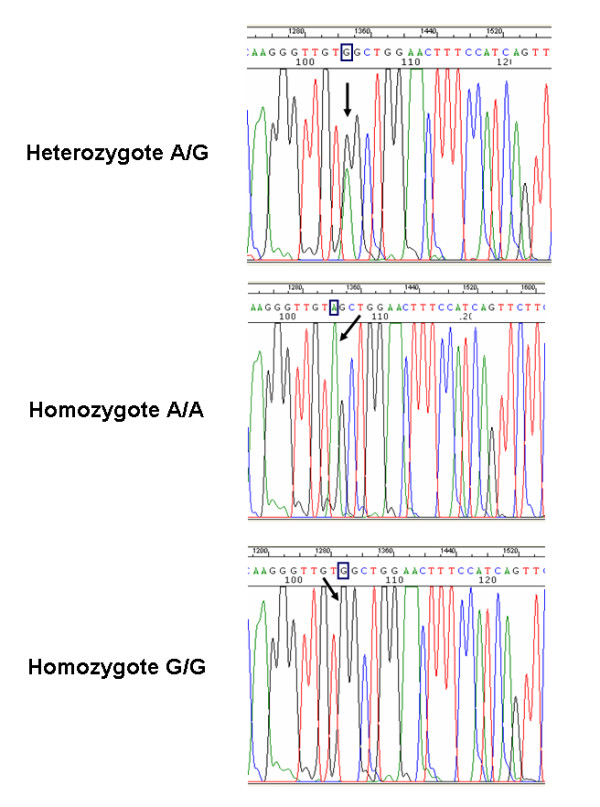
**Sequencing results for the single nucleotide polymorphism at position 61 of the *EGF *gene**. Electropherograms show the nucleotide sequences corresponding to the three different genotypes; arrows indicate the nucleotide position within the sequence.

Figure 1 shows the nucleotide sequences for the three expected genotypes at position 61 of the *EGF *gene: homozygosity A/A, heterozygosity A/G, homozygosity G/G.

As shown in Table 1, both the G/G genotype and the G allele frequencies did not significantly differ according to the total number of benign nevi. When we compared all these individuals, who were considered as non-melanoma healthy controls, with the melanoma cases, again no difference in *EGF *genotype frequencies was observed between such two groups (Table 2). After adjustment for age and sex, the G/G genotype was not more common among melanoma patients compared with healthy subjects (16.3 and 17.6%, respectively; OR, 0.89; 95% CI, 0.29–1.63). Interestingly, a heterogeneous distribution of the frequencies of the G/G genotype was detected among cases and controls of different geographical origin. A non-significant higher prevalence of the G/G genotype was found among melanoma patients as compared with healthy subjects originating from North Italy (21.1 and 18.3%, respectively; OR, 1.39; 95% CI, 0.38–2.77), whereas an inverse distribution was observed among melanoma patients as compared with healthy subjects originating from South Italy (12.6 and 17.1%, respectively; OR, 0.63; 95% CI, 0.22–1.46) (Table 3). Furthermore, there was no evidence that both the G/G genotype and the G allele were associated with disease progression [in terms of Breslow depth (median thickness: A/A genotype, 0.89 mm; G/G genotype, 0.95 mm) and Clark level of invasion (the G/G genotype was found in about 17% of Clark I–II cases, 16% of Clark III cases, and 16% of Clark IV–V cases)] as well as with presence of ulceration as prognostic factor.

**Table 1:** Prevalence of *EGF *genotypes and allele frequencies according to total number of nevi.

**Genotype**	**<10 total nevi** **(%)**	**>100 total nevi** **(%)**
**A/A**	48 (38%)	45 (35%)
**A/G**	56 (44%)	61 (48%)
**G/G**	23 (18%)	22 (17%)
**Total cases**	**127**	**128**
*Allele*		
*A*	*152* *(60%)*	*151* *(59%)*
*G*	*102* *(40%)*	*105* *(41%)*

**Table 2:** Prevalence of *EGF *genotypes and allele frequencies among melanoma and non-melanoma subjects.

**Genotype**	**Healthy controls** **(%)**	**Melanoma patients** **(%)**	Odds ratio* (95% CI)
**A/A**	93 (36.5%)	144 (34.4%)	1.00
**A/G**	117 (45.9%)	206 (49.3%)	1.13 (0.57–2.11)
**G/G**	45 (17.6%)	68 (16.3%)	0.89 (0.29–1.63)
**Total cases**	**255**	**418**	
*Allele*			
*A*	*303* *(60%)*	*494* *(59%)*	*1.00*
*G*	*207* *(40%)*	*342* *(41%)*	*1.04* *(0.39–1.97)*

*adjusted for age and sex. CI, confidence interval

**Table 3:** Distribution of *EGF *genotypes and allele frequencies in Italy among melanoma and non-melanoma subjects.

**Genotype**	**Healthy controls (%)**	**Melanoma patients (%)**	Odds ratio* (95% CI)
North Italy			
**A/A**	45 (35.7%)	51 (28.3%)	1.00
**A/G**	58 (46.0%)	91 (50.6%)	1.26 (0.58–2.43)
**G/G**	23 (18.3%)	38 (21.1%)	1.39 (0.38–2.77)
**Total cases**	**126**	**180**	
*Allele*			
*A*	*148* *(59%)*	*193* *(54%)*	*1.00*
*G*	*104* *(41%)*	*167* *(46%)*	*1.28* *(0.63–2.14)*

South Italy			
**A/A**	48 (37.2%)	93 (39.1%)	1.00
**A/G**	59 (45.7%)	115 (48.3%)	1.02 (0.36–1.98)
**G/G**	22 (17.1%)	30 (12.6%)	0.63 (0.22–1.46)
**Total cases**	**129**	**238**	
*Allele*			
*A*	*155* *(60%)*	*301* *(63%)*	*1.00*
*G*	*103* *(40%)*	*175* *(37%)*	*0.84* *(0.31–1.55)*

*adjusted for age and sex. CI, confidence interval

Our findings seem to suggest that the role of genetic factors in predisposition and/or pathogenesis of melanoma needs to be evaluated in every different populations and geographical areas. Although the absence of impact of the *EGF *+61A>G polymorphism on either development of nevi or melanoma susceptibility in Italy is consistent with data reported in other populations [3-7], an evident discrepancy in prevalence distribution of such a gene sequence variant was observed among cases originating from northern and southern Italian regions. This phenomenon may probably due to a different origin and/or "genetic background" of italian melanoma patients. As for mutation frequencies of other candidate melanoma genes [9], these data are further supporting the hypothesis that genetic factors associated to such a disease may be differently prevalent in North and South Italy, somehow varying according to the variation of the population incidence rates of the disease within these two geographical areas (roughly, 12 versus 4 new melanoma cases per 100.000 inhabitants per year in North Italy and South Italy, respectively [10]). Finally, our study provides evidence against the conclusions of previous reports which indicated that the *EGF *+61A>G polymorphism may be a potential marker for disease severity, predicting a poorer survival [5-7]. This represents a further confirmation that genetic factors modifying melanoma progression might also be geographically heterogeneous.

## Conclusion

The *EGF *+61A>G polymorphism seems to play a limited role on either predisposition or pathogenesis of melanoma; prevalence of such a genetic variant may deeply vary in different populations.

## Competing interests

The authors declare that they have no competing interests.

## Authors' contributions

MC performed mutation analysis. MA participated in design of the study and interpretation of data. MAP participated to collection of cases and controls (from North Italy). RB participated to collection of cases and controls (from Middle Italy). PAA participated to collection of cases and controls (from South Italy). IS participated to data management. SC participated to analysis of data. GrP performed some mutation analyses. EZ performed statistical analysis. GiP conceived of the study and drafted the manuscript.

All authors read and approved the final manuscript.

## Pre-publication history

The pre-publication history for this paper can be accessed here:


